# Psychosis Endophenotypes: A Gene-Set-Specific Polygenic Risk Score Analysis

**DOI:** 10.1093/schbul/sbad088

**Published:** 2023-08-14

**Authors:** Baihan Wang, Haritz Irizar, Johan H Thygesen, Eirini Zartaloudi, Isabelle Austin-Zimmerman, Anjali Bhat, Jasmine Harju-Seppänen, Oliver Pain, Nick Bass, Vasiliki Gkofa, Behrooz Z Alizadeh, Therese van Amelsvoort, Maria J Arranz, Stephan Bender, Wiepke Cahn, Maria Stella Calafato, Benedicto Crespo-Facorro, Marta Di Forti, Genetic Risk and Outcome of Psychosis (GROUP) Study, Ina Giegling, Lieuwe de Haan, Jeremy Hall, Mei-Hua Hall, Neeltje van Haren, Conrad Iyegbe, René S Kahn, Eugenia Kravariti, Stephen M Lawrie, Kuang Lin, Jurjen J Luykx, Ignacio Mata, Colm McDonald, Andrew M McIntosh, Robin M Murray, Psychosis Endophenotypes International Consortium (PEIC), Marco Picchioni, John Powell, Diana P Prata, Dan Rujescu, Bart P F Rutten, Madiha Shaikh, Claudia J P Simons, Timothea Toulopoulou, Matthias Weisbrod, Ruud van Winkel, Karoline Kuchenbaecker, Andrew McQuillin, Elvira Bramon

**Affiliations:** Department of Mental Health Neuroscience, Division of Psychiatry, University College London, London, UK; Nuffield Department of Population Health, University of Oxford, Oxford, UK; Department of Mental Health Neuroscience, Division of Psychiatry, University College London, London, UK; Department of Genetics and Genomic Sciences, Icahn School of Medicine at Mount Sinai, New York, NY, USA; Department of Mental Health Neuroscience, Division of Psychiatry, University College London, London, UK; Institute of Health Informatics, University College London, London, UK; Department of Mental Health Neuroscience, Division of Psychiatry, University College London, London, UK; Department of Mental Health Neuroscience, Division of Psychiatry, University College London, London, UK; Social, Genetic and Developmental Psychiatry Centre, Institute of Psychiatry, Psychology and Neuroscience, King’s College London, London, UK; Department of Mental Health Neuroscience, Division of Psychiatry, University College London, London, UK; Department of Mental Health Neuroscience, Division of Psychiatry, University College London, London, UK; Department of Basic and Clinical Neuroscience, Maurice Wohl Clinical Neuroscience Institute, Institute of Psychiatry, Psychology and Neuroscience, King’s College London, London, UK; Department of Mental Health Neuroscience, Division of Psychiatry, University College London, London, UK; Department of Mental Health Neuroscience, Division of Psychiatry, University College London, London, UK; University of Groningen, University Medical Center Groningen, University Center for Psychiatry, Rob Giel Research Center, Groningen, The Netherlands; Department of Epidemiology, University Medical Center Groningen, Groningen, The Netherlands; Department of Psychiatry and Neuropsychology, School for Mental Health and Neuroscience, Maastricht University Medical Center, Maastricht, The Netherlands; Fundació Docència i Recerca Mutua Terrassa, Terrassa, Spain; Centro de Investigación Biomédica en Red de Salud Mental (CIBERSAM), Institut de Recerca Biomédica Sant Pau (IIB-Sant Pau), Barcelona, Spain; Department of Child and Adolescent Psychiatry, Psychosomatic Medicine and Psychotherapy, Faculty of Medicine and University Hospital Cologne, University of Cologne, Cologne, Germany; Department of Psychiatry, Brain Centre Rudolf Magnus, University Medical Center Utrecht, Utrecht University, Utrecht, The Netherlands; Altrecht, General Mental Health Care, Utrecht, The Netherlands; Department of Mental Health Neuroscience, Division of Psychiatry, University College London, London, UK; CIBERSAM, Centro Investigación Biomédica en Red Salud Mental, Sevilla, Spain; Department of Psychiatry, University Hospital Virgen del Rocio, School of Medicine, University of Sevilla–IBiS, Sevilla, Spain; Social, Genetic and Developmental Psychiatry Centre, Institute of Psychiatry, Psychology and Neuroscience, King’s College London, London, UK; Comprehensive Centers for Clinical Neurosciences and Mental Health (C3NMH), Medical University of Vienna, Austria; Department of Psychiatry, Amsterdam UMC, University of Amsterdam, Amsterdam, The Netherlands; Arkin, Institute for Mental Health, Amsterdam, The Netherlands; Neuroscience and Mental Health Innovation Institute, School of Medicine, Cardiff University, Hadyn Ellis Building, Mandy Road, Cardiff, UK; Psychosis Neurobiology Laboratory, Harvard Medical School, McLean Hospital, Belmont, MA, USA; Department of Child and Adolescent Psychiatry/Psychology, Erasmus University Medical Center, Sophia’s Children Hospital, Rotterdam, The Netherlands; Department of Genetics and Genomic Sciences, Icahn School of Medicine at Mount Sinai, New York, NY, USA; Department of Psychiatry, Icahn School of Medicine at Mount Sinai, New York, NY, USA; Institute of Psychiatry, Psychology and Neuroscience, King’s College London, London, UK; Division of Psychiatry, University of Edinburgh, Royal Edinburgh Hospital, Edinburgh, UK; Nuffield Department of Population Health, University of Oxford, Oxford, UK; Department of Psychiatry, Brain Centre Rudolf Magnus, University Medical Center Utrecht, Utrecht University, Utrecht, The Netherlands; Department of Translational Neuroscience, Brain Center Rudolf Magnus, University Medical Center Utrecht, Utrecht, The Netherlands; Fundacion Argibide, Pamplona, Spain; CIBERSAM, Centro Investigación Biomédica en Red Salud Mental, Madrid, Spain; The Centre for Neuroimaging & Cognitive Genomics (NICOG) and NCBES Galway Neuroscience Centre, University of Galway, Galway, Ireland; Division of Psychiatry, University of Edinburgh, Royal Edinburgh Hospital, Edinburgh, UK; Centre for Cognitive Ageing and Cognitive Epidemiology, University of Edinburgh, Edinburgh, UK; Institute of Psychiatry, Psychology and Neuroscience, King’s College London, London, UK; Institute of Psychiatry, Psychology and Neuroscience, King’s College London, London, UK; St Magnus Hospital, Surrey, UK; Institute of Psychiatry, Psychology and Neuroscience, King’s College London, London, UK; Institute of Psychiatry, Psychology and Neuroscience, King’s College London, London, UK; Instituto de Biofísica e Engenharia Biomédica, Faculdade de Ciencias da Universidade de Lisboa, Portugal; Department of Psychiatry, Amsterdam UMC, University of Amsterdam, Amsterdam, The Netherlands; Division of General Psychiatry, Medical University of Vienna, Austria; Department of Psychiatry and Neuropsychology, School for Mental Health and Neuroscience, Maastricht University Medical Center, Maastricht, The Netherlands; North East London Foundation Trust, London, UK; Research Department of Clinical, Educational and Health Psychology, University College London, London, UK; Department of Psychiatry and Neuropsychology, School for Mental Health and Neuroscience, Maastricht University Medical Center, Maastricht, The Netherlands; GGzE Institute for Mental Health Care, Eindhoven, The Netherlands; Institute of Psychiatry, Psychology and Neuroscience, King’s College London, London, UK; Interdisciplinary Program in Neuroscience, Aysel Sabuncu Brain Research Center, Bilkent University, Ankara, Türkiye; National Magnetic Resonance Research Center (UMRAM), Bilkent University, Ankara, Türkiye; Department of Psychology, Bilkent University, Ankara, Türkiye; School of Medicine, Department of Psychiatry, National and Kapodistrian University of Athens, Athens, Greece; Department of Psychiatry and Behavioral Health System, Icahn School of Medicine at Mount Sinai, New York, USA; Department of General Psychiatry, Center of Psychosocial Medicine, University of Heidelberg, Germany; SRH Klinikum, Karlsbad-Langensteinbach, Germany; Department of Psychiatry and Neuropsychology, School for Mental Health and Neuroscience, Maastricht University Medical Center, Maastricht, The Netherlands; KU Leuven, Department of Neuroscience, Research Group Psychiatry, Leuven, Belgium; Department of Mental Health Neuroscience, Division of Psychiatry, University College London, London, UK; UCL Genetics Institute, Division of Biosciences, University College London, London, UK; Department of Mental Health Neuroscience, Division of Psychiatry, University College London, London, UK; Department of Mental Health Neuroscience, Division of Psychiatry, University College London, London, UK; Institute of Cognitive Neuroscience, University College London, London, UK

**Keywords:** schizophrenia, bipolar disorder, EEG, P300, neurodevelopment

## Abstract

**Background and Hypothesis:**

Endophenotypes can help to bridge the gap between psychosis and its genetic predispositions, but their underlying mechanisms remain largely unknown. This study aims to identify biological mechanisms that are relevant to the endophenotypes for psychosis, by partitioning polygenic risk scores into specific gene sets and testing their associations with endophenotypes.

**Study Design:**

We computed polygenic risk scores for schizophrenia and bipolar disorder restricted to brain-related gene sets retrieved from public databases and previous publications. Three hundred and seventy-eight gene-set-specific polygenic risk scores were generated for 4506 participants. Seven endophenotypes were also measured in the sample. Linear mixed-effects models were fitted to test associations between each endophenotype and each gene-set-specific polygenic risk score.

**Study Results:**

After correction for multiple testing, we found that a reduced P300 amplitude was associated with a higher schizophrenia polygenic risk score of the forebrain regionalization gene set (mean difference per *SD* increase in the polygenic risk score: −1.15 µV; 95% CI: −1.70 to −0.59 µV; *P* = 6 × 10^−5^). The schizophrenia polygenic risk score of forebrain regionalization also explained more variance of the P300 amplitude (*R*^2^ = 0.032) than other polygenic risk scores, including the genome-wide polygenic risk scores.

**Conclusions:**

Our finding on reduced P300 amplitudes suggests that certain genetic variants alter early brain development thereby increasing schizophrenia risk years later. Gene-set-specific polygenic risk scores are a useful tool to elucidate biological mechanisms of psychosis and endophenotypes, offering leads for experimental validation in cellular and animal models.

## Introduction

Psychotic disorders are highly heritable, with a heritability estimate of approximately 80% for schizophrenia and bipolar disorder.^1,2^ Breakthroughs have been made by genome-wide association studies (GWAS) in understanding the genetic basis of psychosis, with 270 loci associated with schizophrenia and 64 loci associated with bipolar disorder identified so far.^3,4^ Although these findings are promising, the functional effects of these variants in the pathophysiology of psychosis are still in the process of being understood.

Partitioning the effects of risk loci into distinct brain functional domains can provide important biological insights into the mechanisms of psychosis. One such approach uses endophenotypes, ie, heritable phenotypes associated with a, putatively more complex, illness.^5^ As such, a biomarker is considered an endophenotype if it is heritable and consistently shown to be altered in both patients and their unaffected relatives.^6^ Previous studies have established several endophenotypes for psychosis, such as verbal memory,^7,8^ executive functions,^9^ P300 amplitudes/latencies,^10–13^ and lateral ventricular volumes.^14^

Polygenic risk scores, the sum of the number of risk alleles weighted by their effect sizes, provide a method to test the genetic overlap between psychosis and its endophenotypes. However, previous studies testing associations between the polygenic risk scores for schizophrenia/bipolar disorder and psychosis endophenotypes yielded mixed results.^15–21^ This could be because genome-wide polygenic risk scores combine many risk alleles across the genome, but only a subset of them are associated with an endophenotype related to a specific biological process.^21^

Gene-set-specific polygenic risk scores can be a useful tool to address the issue. They are the effect size-weighted sum of risk alleles restricted to genes within a particular gene set (often associated with a biological process), thus only containing a subset of risk alleles that might be relevant to a specific endophenotype. For instance, in a sample of 333 participants, Rampino et al found that both attentional performance and prefrontal cortex activity during an attention control task were associated with the schizophrenia polygenic risk score of glutamate signaling.^22^ Merikanto et al calculated a schizophrenia polygenic risk score for the CACNA1l region and found that it was significantly associated with sleep spindle amplitude, duration, and intensity in a sample of 157 adolescents.^23^ By contrast, 2 studies with 167 to 2725 participants did not find an association between gene-set-specific schizophrenia polygenic risk scores related to neurotransmission/neurodevelopment and brain volumes measured by magnetic resonance imaging (MRI).^24,25^

In summary, the utility of gene-set-specific polygenic risk scores needs further testing in a broader range of psychosis endophenotypes. More gene sets should be studied, as previous studies only focused on a small number of hypothesis-driven gene sets. Therefore, by testing the association between 7 known psychosis endophenotypes and gene-set-specific polygenic risk scores for schizophrenia and bipolar disorder, the current study aims to identify the biological processes underlying the genetic risk for psychosis.

## Methods

### Participants and Clinical Assessments

Overall, 6935 participants were recruited by the Psychosis Endophenotypes International Consortium (PEIC) at 8 research centers in Australia, Germany, the Netherlands (as part of the Genetic Risk and Outcome of Psychosis [GROUP] Study), Spain, and the United Kingdom. The study was approved by the local ethics committee at each research center. All participants provided written informed consent before assessments. There were 3 clinical groups recruited in the sample: Patients with psychosis, their unaffected first-degree relatives, and controls. Diagnoses were made based on the Diagnostic and Statistical Manual of Mental Disorders, fourth edition (DSM-IV)^26^ and structured clinical interviews.^27–32^ Details of diagnostic measures and inclusion and exclusion criteria can be found in supplementary materials.

### Cognitive Measures

Participants were assessed by the block design and digit span tasks in the Wechsler Adult Intelligence Scale, revised version (WAIS-R)^33^ or third edition (WAIS-III).^34^ The block design task measured participants’ visuospatial ability and the digit span task measured participants’ short-term and working memory. As different research centers adopted slightly different versions of the block design and digit span tasks, we used percentage (raw score/max score) to represent participants’ performance in the 2 tasks. Participants were also assessed by the Rey Auditory Verbal Learning Test,^35,36^ which included the immediate and delayed recall tests (measuring short-term and long-term verbal memory).

### EEG and MRI Data Collection and Processing

The P300 was measured using the auditory oddball task at 3 research centers, during which participants listened to a series of high-pitched target/deviant tones (10%–20%) randomly embedded in many low-pitched non-target/standard tones (80%–90%).^11,37–40^ EEG data were collected with vertical electrooculography (EOG) from 17 to 20 scalp sites based on the International 10/20 system,^41^ referenced to mastoids or earlobes. EEG was corrected for eye blink artifacts using regression-based weighting coefficients,^42^ as well as additional visual inspection. The P300 amplitude and latency were measured at the peak between 250 and 600 ms following the target tones at the Pz electrode. Lateral ventricular volumes were measured at 5 research centers by MRI, which included the body and the frontal, occipital, and temporal horns.^43–58^

### Genotyping, Quality Control, and Imputation

Blood DNA samples of 6935 participants were collected at all research centers and sent to the Wellcome Trust Sanger Institute (Cambridge, UK) for initial processing and quality control. Subsequently, samples were sent to Affymetrix Services Laboratory (www.affymetrix.com) for genotyping. Genotypes were called using the CHIAMO algorithm modified for use with the Affymetrix 6.0 genotyping array.^59,60^ They underwent standard quality control at UCL using software including PEDSTATS,^61^ Evoker,^62^ LDAK,^63^ and PLINK.^64^ Quality-controlled genotypes were uploaded to the Sanger Imputation Server (https://imputation.sanger.ac.uk) for imputation.^65^ Pre-phasing and imputation were conducted according to the EAGLE2/PWBT pipeline based on the Haplotype Reference Consortium panel (r1.1).^66,67^ The imputed genotypes were converted to best-guess format using a hard-call threshold of 0.8 and SNPs with an INFO score <0.8 were excluded. A total of 6 215 801 SNPs and 4835 participants remained after quality control. Details of genotyping, quality control, and imputation can be found in supplementary materials and previous publications.^17,68–70^

### Relationship Inference and Principal Component Analysis

To account for familial relatedness and population structure in the sample, we used the GENESIS R/Bioconductor package to generate a kinship matrix and conduct principal component (PC) analysis.^71,72^ Based on the genotyped data that passed quality control, an unadjusted kinship matrix was first generated using KING-robust 2.2.5.^73^ The genotyped data were further pruned using the SNPRelate package in R 4.0.2^74^ and analyzed with the unadjusted kinship matrix by the PC-AiR function to estimate the ancestrally representative PCs.^71^ We then estimated a new kinship matrix adjusted for the PCs by the PC-Relate function, which allows for more accurate estimation of familial relatedness independent of ancestral background.^75^ Details of relationship inference and PC analysis can be found in supplementary materials.

### Selection of Gene Sets

We retrieved a group of gene sets related to the central nervous system from previous publications,^76–78^ most of which were derived from the Mouse Genome Informatics Mammalian Phenotype database.^79^ We downloaded other lists of curated gene sets from the following public access databases: Reactome,^80^ Kyoto Encyclopedia of Genes and Genomes,^81^ Pathway Commons,^82^ and Panther.^83^ Gene sets from the “Cellular Component” and “Biological Process” categories were downloaded from Gene Ontology.^84^ To reduce the burden of multiple testing correction, for gene sets downloaded from public databases we retained only those with at least one of the following key terms: Brain, cerebral, nerve, nervous, neuron, neuronal, neural, glia, microglia, astrocyte, oligodendrocyte, axon, axonal, dendrite, dendritic, synapse, synaptic, neurotransmitter, or neurotransmission. Gene sets with terms indicating the direction of regulation (ie, positive or negative) were removed, as gene sets were only used to subset SNPs and the direction of regulation of the gene sets would not be relevant to polygenic risk scores. Based on these criteria, we included a total of 378 gene sets in our final analysis.

### Polygenic Risk Scoring

We used PRSice v2.3.3^85,86^ to calculate the genome-wide polygenic risk scores for schizophrenia and bipolar disorder for each individual in the PEIC sample. GWAS summary statistics for schizophrenia and bipolar disorder were downloaded from the Psychiatric Genomics Consortium (PGC3).^3,4^ As the PEIC sample only included participants of European ancestry and was part of the PGC3 sample, the GWAS summary statistics we used were generated based on the European participants of the PGC3 that excluded the PEIC sample. We excluded SNPs with an INFO score <0.8 or a minor allele frequency <0.01 (in cases or controls) in the GWAS summary statistics, and performed clumping with an r^2^ threshold = 0.1 in a 500 kilobase window. We applied a *P*-value threshold of 1 to include all SNPs that passed the quality control to calculate the genome-wide schizophrenia and bipolar disorder polygenic risk scores. We also applied a *P*-value threshold of .05 for the genome-wide schizophrenia polygenic risk score and 0.1 for the genome-wide bipolar disorder polygenic risk score, as those *P*-value thresholds generated the polygenic risk scores that explained the most variance in disease risk in the previous publications by the PGC3.^3,4^

We then used the PRSet function in PRSice v2.3.3 to calculate the gene-set-specific polygenic risk scores.^85,87^ Compared to other methods,^88,89^ PRSet is computationally efficient and performs clumping for each gene set to keep all independent signals.^87^ We calculated the scores of each gene set selected above for schizophrenia and bipolar disorder separately. The method used here was similar to that for the genome-wide polygenic risk scores, but restricted to SNPs that fall within a 10-kilobase window around each gene included in a gene set. SNPs were clumped independently for each gene set using an *r*^2^ threshold = 0.1 in a 2-megabase window. We applied a *P*-value threshold of 1 for all gene-set-specific polygenic risk scores without excluding any SNPs after clumping, to maximize the number of SNPs included in each gene set.

In total, we generated 380 (378 gene-set specific, 2 genome-wide) polygenic risk scores for schizophrenia and 378 (376 gene-set specific, 2 genome-wide) polygenic risk scores for bipolar disorder. Two gene sets were excluded from the bipolar disorder polygenic risk scores as no SNPs in the gene sets were found in the GWAS summary statistics and the PEIC sample.

### Statistical Analysis

Our primary analysis tested associations between the 7 endophenotypes and the polygenic risk scores. We standardized the polygenic risk scores based on the means and SDs of the control group. For each endophenotype, we fitted a linear mixed-effects regression model with each polygenic risk score as a fixed effect. For covariates, we included age, sex, clinical group, research center, and the first 4 ancestry PCs as fixed effects, and the kinship matrix as a random effect. For significant associations, we also checked if the associations were consistent across 3 clinical groups and if they were driven by specific genes in the gene set.

In our secondary analysis, we tested associations between the polygenic risk scores and participants’ case–control status, including only patients and controls. We fitted a fixed-effect logistic regression model with case–control status as a binary outcome and each of the gene-set-specific polygenic risk scores as a fixed effect. We included age, sex, research center, and the first 4 ancestry PCs in the model as covariates. The kinship matrix was not included as participants in the patient and control groups were generally unrelated. Participants recruited in Munich or Pamplona were excluded from the analysis as the 2 centers recruited only patients or only controls.

We accounted for multiple testing using Bonferroni correction, generating a new significance threshold based on the number of polygenic risk scores tested for each endophenotype (0.05/(380 + 378) = 7 × 10^−5^), and additionally applied a more stringent threshold accounting for the number of endophenotypes (0.05/(380 + 378)/7 = 9 × 10^−6^). We used Nakagawa’s *R*^2^ to indicate the variance of each endophenotype explained by each polygenic risk score,^90^ and Nagelkerke’s pseudo *R*^2^ for case–control status to indicate the improvement of the model by adding the polygenic risk score compared to the null model without it.^91^ We initially included an interaction term between polygenic risk score and clinical group in the model, but eventually dropped it as no significant interactions were detected after correction for multiple testing.

For all analyses mentioned above, we excluded participants who did not pass genetic quality control or with missing data on any of the covariates included in the model. As different research centers collected different endophenotypes, the total number of participants analyzed in the models also varied across endophenotypes. All statistical analyses were conducted using R 4.0.2.^72^

## Results

### Overview

Polygenic risk scores were calculated for 4835 participants that passed genetic quality control. After excluding participants with missing data on relevant covariates, there were 4506 participants left for further analysis. Of the 4506 participants, there were 1182 (26%) patients, 854 (19%) unaffected relatives, and 2470 (55%) controls, and the mean age of the sample was 42.4 (*SD* = 15.8) years, with 2186 (49%) females and 2320 (51%) males. Among the patients, there were 906 (77%) diagnosed with schizophrenia, 107 (9%) with bipolar disorder, and 169 (14%) with other psychotic disorders. table 1 shows detailed information on sample characteristics by clinical group.

**Table 1.** Sample Characteristics by Clinical Group

Variable	Patient (*n* = 1182)	Relative (*n* = 854)	Control (*n* = 2470)	Total (*n* = 4506)
Mean (SD) age (years)	33.5 (10.4)	45.7 (15.9)	45.5 (16.2)	42.4 (15.8)
Sex				
Female	388 (33%)	510 (60%)	1288 (52%)	2186 (49%)
Male	794 (67%)	344 (40%)	1182 (48%)	2320 (51%)
Diagnosis				
Schizophrenia	906 (77%)	0 (0%)	0 (0%)	906 (20%)
Bipolar disorder	107 (9%)	0 (0%)	0 (0%)	107 (2%)
Other psychotic disorder	169 (14%)	0 (0%)	0 (0%)	169 (4%)
Depressive disorder	0 (0%)	156 (18%)	158 (6%)	314 (7%)
Anxiety disorder	0 (0%)	27 (3%)	12 (1%)	39 (1%)
Substance misuse	0 (0%)	4 (1%)	11 (0%)	15 (0%)
Anxiety and depressive disorder	0 (0%)	9 (1%)	3 (0%)	12 (0%)
Personality disorder	0 (0%)	1 (0%)	0 (0%)	1 (0%)
No Psychiatric disorders	0 (0%)	657 (77%)	2,286 (93%)	2,943 (65%)
Research center				
Edinburgh	31 (3%)	0 (0%)	17 (1%)	48 (1%)
Heidelberg	24 (2%)	9 (1%)	22 (1%)	55 (1%)
London	237 (20%)	197 (23%)	324 (13%)	758 (17%)
Munich	0 (0%)	0 (0%)	962 (39%)	962 (21%)
The Netherlands	370 (31%)	505 (59%)	974 (39%)	1,849 (41%)
Pamplona	44 (4%)	0 (0%)	0 (0%)	44 (1%)
Perth	309 (26%)	143 (17%)	163 (7%)	615 (14%)
Santander	167 (14%)	0 (0%)	8 (0%)	175 (4%)

*Note.* The Netherlands included 4 study sites (Amsterdam, Groningen, Maastricht, and Utrecht) in the GROUP Study, which employed similar recruitment and assessment procedures.

The summary statistics of the 7 endophenotype measures by clinical group are shown in table 2, and the sample sizes vary across different endophenotypes (*n* = 510 to 3088). In general, patients and relatives showed deficits in all endophenotypes compared to controls, which has been reported in our previous publications using the same sample.^17,68,69^

**Table 2.** Summary Statistics of Endophenotype Measures by Clinical Group

Endophenotype	Patient		Relative		Control		Total	
	n	mean (SD)	n	mean (SD)	n	mean (SD)	n	mean (SD)
Block design (%)	488	54.0 (28.0)	592	51.5 (28.0)	2008	60.0 (21.4)	3088	57.4 (23.8)
Digit span (%)	263	47.5 (14.2)	58	41.4 (13.4)	1116	51.5 (14.6)	1437	50.4 (14.7)
Lateral ventricular volume (cm^3^)	322	17.1 (10.3)	174	18.2 (11.5)	279	15.5 (8.8)	775	17.1 (16.8)
P300 amplitude (μV)	211	10.8 (6.1)	160	12.1 (7.5)	139	13.4 (6.8)	510	11.9 (6.8)
P300 latency (ms)	212	382.3 (53.1)	164	386.5 (55.5)	139	358.2 (38.0)	515	377.2 (51.6)
RAVLT immediate recall score	633	21.9 (6.3)	621	25.2 (6.3)	964	26.0 (6.1)	2218	24.6 (6.4)
RAVLT delayed recall score	629	6.7 (3.1)	617	8.5 (2.9)	950	8.7 (2.8)	2196	8.1 (3.1)

*Note.* Participants’ performance in the block design and digit span tasks was measured by percentage (raw score/max score). RAVLT, Rey Auditory Verbal Learning Test.

### Associations Between Endophenotypes and Polygenic Risk Scores

**Fig. 1.**
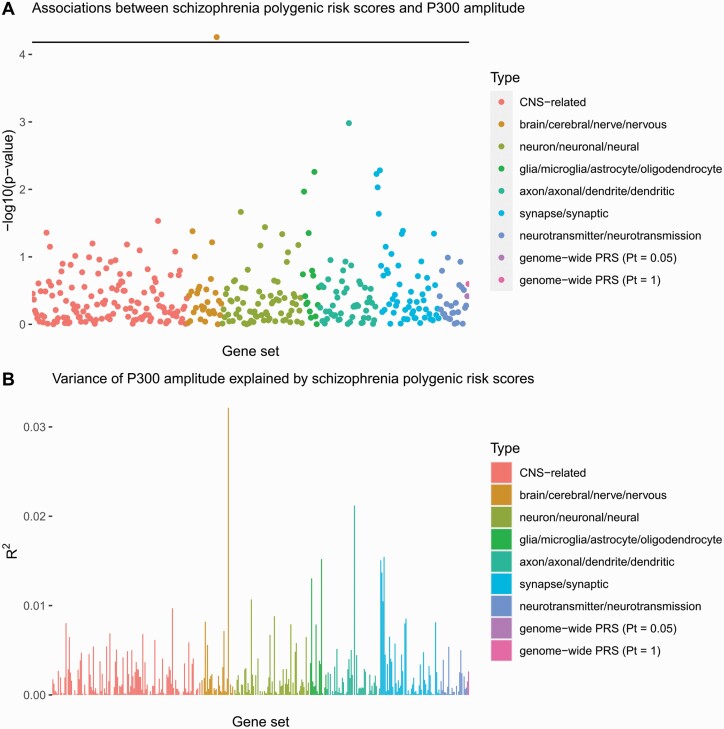
Associations between P300 amplitude and schizophrenia polygenic risk scores (A) and variance of P300 amplitude explained by schizophrenia polygenic risk scores (B). Gene-set-specific polygenic risk scores are grouped by the search terms they contain. CNS-related polygenic risk scores were generated based on custom annotated gene sets from previous publications.^76–78^ On the x-axis, gene sets from the same source were arranged in descending order of the number of SNPs included in each polygenic risk score. CNS, central nervous system; PRS, polygenic risk score; Pt, *P*-value threshold.

Based on the significance threshold of 7 × 10^−5^ after multiple testing corrections, we found a significant negative association between the P300 amplitude and the schizophrenia polygenic risk score of forebrain regionalization in a sample of 510 participants (211 patients, 160 relatives, and 139 controls; mean difference per SD increase in the polygenic risk score: −1.15 µV; 95% CI: −1.70 to −0.59 µV; *P* = 6 × 10^−5^; figure 1A). The schizophrenia polygenic risk score of forebrain regionalization also explained more variance of the P300 amplitude (*R*^2^ = 0.032) than any other schizophrenia polygenic risk scores, including the genome-wide schizophrenia polygenic risk scores with a *P*-value threshold of 0.05 (*R*^2^ = 0.015) and 1 (*R*^2^ = 0.019) (figure 1B).

As validation, we also checked if the association between the P300 amplitude and the schizophrenia polygenic risk score of forebrain regionalization was consistent across 3 clinical groups. The direction of the association was consistent in all groups, which also reached the nominal significance level (*P* < .05) in both patients and controls (supplementary figure S5). Notably, *EMX1*, one of the genes within the forebrain regionalization gene set, contained a locus that reached genome-wide significance in the latest GWAS on schizophrenia.^4^ Indeed, an additional analysis showed that higher partitioned schizophrenia polygenic risk scores restricted to the EMX1 region were associated with reduced P300 amplitudes at the nominal significance level (mean difference per SD increase in polygenic risk score: −0.66 µV, 95% CI: −1.27 to −0.05, *P* = .033) (supplementary materials).

No significant associations were found between other endophenotypes and schizophrenia or bipolar disorder polygenic risk scores after correction for multiple testing (supplementary figure S1 to S4). The −log10(*P*-value) for those associations was not or very weakly correlated with the number of SNPs included in the polygenic risk scores, indicating that our results were not confounded by the number of SNPs in each score (supplementary materials). No associations passed the more stringent significance threshold of 9 × 10^−6^.

### Associations Between Case–Control Status and Polygenic Risk Scores

**Fig. 2.**
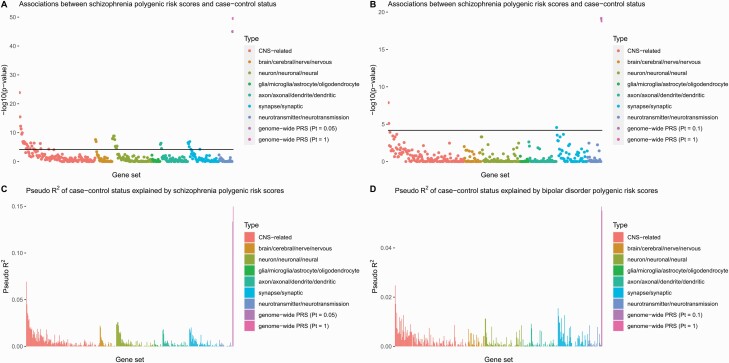
Associations between case–control status and schizophrenia (A) or bipolar disorder (B) polygenic risk scores. Pseudo R^2^ of case–control status explained by schizophrenia (C) or bipolar disorder (D) polygenic risk scores. Gene-set-specific polygenic risk scores are grouped by the search terms they contain. CNS-related polygenic risk scores were generated based on custom annotated gene sets from previous publications.^76–78^ On the x-axis, gene sets from the same source were arranged in descending order of the number of SNPs included in each polygenic risk score. CNS, central nervous system; PRS, polygenic risk score; Pt, *P*-value threshold.

For associations with case–control status in a sample of 1138 cases and 1508 controls, 55 gene-set specific polygenic risk scores for schizophrenia and 18 gene-set specific polygenic risk scores for bipolar disorder passed the 7 × 10^−5^ threshold after multiple testing corrections. However, the genome-wide polygenic risk scores were generally more significantly associated than the gene-set-specific polygenic risk scores (figure 2A and figure 2B). The genome-wide polygenic risk scores also had a much bigger pseudo *R*^2^ than any of the gene-set specific polygenic risk scores, as shown in figure 2C and figure 2D. In general, stronger associations with case–control status were found for polygenic risk scores that included more SNPs (supplementary materials).

## Discussion

The current study used gene-set-specific polygenic risk scores as a tool to investigate the biological mechanisms underlying endophenotypes that convey psychosis risk. A significant association was found between the P300 amplitude and the schizophrenia gene-set-specific polygenic risk score of forebrain regionalization. The reduction in P300 amplitudes is a well-established endophenotype for psychosis,^10–13^ and may predict transition to psychosis in individuals at ultra-high risk.^92,93^ However, no compelling theories have been developed to explain the underlying neurobiology of P300 deficits in schizophrenia, and our study indicates that they may be related to alterations in early brain development.

Forebrain regionalization is a critical stage in early brain development, during which highly regionalized gene expression modulates the patterning of discrete regions.^94^ This involves several processes such as cell migration and neuronal differentiation, facilitating the separation of the forebrain into the telencephalon (cerebrum) and the diencephalon (thalamus, hypothalamus, epithalamus, and subthalamus).^95^ In line with the finding on the P300 amplitude, a recent transcriptome-wide association study by our group suggests that early neurodevelopment may also influence mismatch negativity, another EEG measure associated with auditory change detection.^70^ Moreover, the role of forebrain development in schizophrenia is supported by a study using human induced pluripotent stem cells (hiPSCs).^96^ In this study, the authors found that genes differentially expressed in neural progenitor cells and neurons between patients with schizophrenia and controls were enriched in the forebrain development pathway.^97^ Interestingly, they found that hiPSC-derived neurons from patients exhibited altered electrophysiological measures related to Na^+^ channel function.^97^ It is plausible that such changes at the neuronal level may also influence higher-level neurophysiological measures such as the P300, although more research is needed to draw this link.

Our additional analysis revealed that the partitioned schizophrenia polygenic risk score restricted to *EMX1* was negatively associated with the P300 amplitude at the nominal *P*-value threshold. This gene contains a genome-wide significant locus identified by the latest schizophrenia GWAS^4^ and is involved in several critical biological processes during early brain development, such as neuron differentiation and neural stem cell proliferation.^98,99^ Thus, given the strong evidence for the involvement of the *EMX1* gene in schizophrenia and in P300 amplitude deficits, further research should seek to characterize its functions using cellular and animal models as well as other endophenotypes in humans.

We found no significant associations for other endophenotypes measured in the current study. This could be explained by the relatively high heritability of the P300 amplitude (69%)^37^ compared to other endophenotypes, such as specific cognitive abilities (average heritability estimates of 56%).^100^ Moreover, the lack of significant associations with bipolar disorder polygenic risk scores might reflect the small number of patients with bipolar disorder in our sample, which limited the statistical power. Finally, it is worth noting that our significant finding did not survive the additional more stringent correction. Therefore, caution needs to be taken when interpreting our results, and future replication studies are needed.

As expected, our secondary analysis revealed that compared to gene-set specific polygenic risk scores, genome-wide polygenic risk scores were more strongly associated with and explained more variance of case–control status. Nevertheless, investigating the associations between gene-set-specific polygenic risk scores and case–control status may still help to pinpoint the core gene sets that are most relevant to disease mechanisms. Although this is beyond the scope of the current study, a previous study found that the schizophrenia polygenic risk scores generated based on predefined core gene sets outperformed polygenic risk scores of randomly generated gene sets of similar sizes.^101^

The present study has its limitations. Although the PEIC has a relatively large sample size, our study might still be underpowered to detect certain associations. More associations between endophenotypes and gene sets may arise in future studies with increased power through meta- or mega-analyses of multiple samples. Moreover, while data from multiple research centers increased the overall sample size, this might have also increased heterogeneity. Nevertheless, we have controlled for potential confounders by including multiple covariates in the regression models, and a strength of this study is that all blood samples underwent the same genotyping and quality control process. Finally, it is worth noting that other factors, such as gene–gene/gene-environment interactions and rare variants associated with psychosis may also influence endophenotypes. Although those were not tested in the current study, our previous study using the same dataset found that schizophrenia-related rare copy number variants were associated with verbal memory deficits.^69^ Certain environmental exposures, such as medication, could also affect endophenotype performance.^102^ Although medication use was not recorded in the PEIC, we believe our finding on the P300 is still valid, as the association was consistent in unaffected relatives and controls who were medication-free (supplementary materials).

To conclude, the current study offered evidence for the utility of endophenotypes and gene-set-specific polygenic risk scores to illuminate the biological mechanisms underlying psychosis. We found that a reduced P300 amplitude was associated with a higher schizophrenia polygenic risk score of forebrain regionalization, supporting the neurodevelopmental hypothesis of schizophrenia.^103,104^ Future studies with larger samples and more gene sets will advance our understanding of biological processes underlying endophenotypes for psychosis. We also need more mechanistic studies, such as those using animal models and human-induced pluripotent stem cells from patients with psychosis, to further illuminate how neurodevelopmental impairments affect endophenotypes and increase psychosis risk.

## Supplementary Material

sbad088_suppl_Supplementary_Materials
