# Supplementary material for: Psychosis Endophenotypes: A Gene-Set-Specific Polygenic Risk Score Analysis
Source: Schizophr Bull. 2023 Aug 14;49(6):1625–36. doi: 10.1093/schbul/sbad088 (PMC10686343; doi:10.1093/schbul/sbad088)
Supplement: sbad088_suppl_Supplementary_Materials [file sbad088_suppl_supplementary_materials.zip › supplementary materials 030523.docx]

Supplementary materials

Participants and clinical assessments

6,935 participants were recruited in the Psychosis Endophenotypes International Consortium (PEIC) at eight research centres in Australia, Germany, the Netherlands (as part of the GROUP project), Spain, and the United Kingdom. The study was approved by the local ethics committee at each research centre. All participants provided written informed consent before assessments.

There were three study groups in the PEIC dataset: patients with psychosis, their unaffected first-degree relatives, and controls. Patients were defined as those who received a diagnosis of psychosis (schizophrenia, bipolar disorder, schizoaffective disorder, schizophreniform disorder, delusional disorder, brief psychotic episode or other psychotic disorder not otherwise specified) based on the Diagnostic and Statistical Manual of Mental Disorders, fourth edition (DSM-IV).^26^ The diagnoses were confirmed by one of the following structured clinical interviews: the Comprehensive Assessment of Symptoms and History (CASH),^27^ the Structured Clinical Interview for DSM Disorders (SCID),^28,29^ the Schedule for Affective Disorders and Schizophrenia (SADS),^30^ the Schedule for Clinical Assessment in Neuropsychiatry, Version 2.0 (SCAN),^31^ or the positive and negative syndrome scale (PANSS) for schizophrenia.^32^ Patients were excluded if they had a history of neurological disorders or a loss of consciousness due to head injury lasting more than a few minutes. Unaffected relatives were defined as those who were the first-degree relatives of the patients without a personal history of psychosis. Controls in the PEIC dataset did not have any personal or family histories of psychosis. To make the sample representative of the general population, relatives and controls with non-psychotic disorders were included as long as they were not on any psychotropic medications in the 12 months preceding study enrolment.

Participants in the PEIC were assessed on seven psychosis endophenotypes across cognitive, brain functional and brain structural domains. They were selected based on previous literature that showed deficits in patients and their unaffected relatives compared to controls. Measurements of the seven endophenotypes are summarised below, and the sample size of endophenotypes varies as they were measured independently by different centres.

Cognitive tests

Participants were assessed by the block design and the digit span tasks in the Wechsler Adult Intelligence Scale, revised version (WAIS-R)^33^ or third edition (WAIS-III).^34^ The block design task measures participants’ visuospatial ability, which asked them to rearrange coloured blocks to match a pattern. The digit span task measured participants’ short-term verbal memory. Participants were asked to recall a sequence of digits in the original order (forward span) or the reverse order (backward span). The digit span score in the PEIC combined the forward and backward span tasks. As different research centres used slightly different versions of the tasks, we used percentage (raw score/max score) to represent participants’ performance in the two tasks. Participants were also assessed by the Rey Auditory Verbal Learning Test (RAVLT).^35,36^ In the RAVLT, participants were firstly verbally presented with a list of 15 words and then asked recall them, and the same procedure was repeated three times in total. The sum of the number of correctly recalled words was recorded as the RAVLT immediate recall score. After 30 minutes of doing other non-verbal cognitive tasks, participants were asked to recall the 15 words again and the number of correctly recalled words was recorded as the RAVLT delayed recall score.

EEG data collection and processing

The P300 was measured using the auditory oddball task at three research centres, during which participants listened to two types of tones: the standard non-target tones, which were low-pitched tones of 1,000 Hz; and the deviant target tones, which were high-pitched tones of 1,500 Hz. In summary, participants listened to 150-800 tones of 80 or 97 dB. Each tone lasted 20 to 50 ms with an inter-stimulus interval of 1000 to 2200 ms. The percentage of the target tones was 10% or 20%. Of the participants, 90% were asked to press a button when the target tone occurred, and 10% were asked to close their eyes to count the number of target tones. Details of the oddball paradigm at each research centre are listed in Table S1 and can be found in previous publications.^11,37–40^

Table S1. Parameters for auditory oddball tasks.

| Research centre | Number of trials | Sound pressure (dB) | Deviant tone (%; Hz) | Standard tone (%; Hz) | Tone duration (ms) | Interstimulus interval (s) | Action required |
| --- | --- | --- | --- | --- | --- | --- | --- |
| Heidelberg | 150 | 97 | 20; 1500 | 80; 1000 | 40 | 1 | Silently count |
| London | 400 | 80 | 20; 1500 | 80; 1000 | 20 | 1.8 – 2.2 | Press a button |
| Perth | 200 * 4 | 80 | 10; 1500 | 90; 1000 | 50 | 1 | Press a button |

EEG data were collected with vertical electrooculography (EOG) from 17 to 20 scalp sites based on the International 10/20 system,^41^ referenced to mastoids or earlobes. Data was digitalized at a sampling rate of 200 to 500 Hz with a low-pass filter of 30 to 120 Hz and/or a high-pass filter of 0.03 or 0.05 Hz. EEG was corrected for eye blink artefacts using regression-based weighting coefficients,^42^ with some of them visually inspected blindly to disease status for other biological artefacts. After baseline correction, the data were averaged for correctly identified target tones, with an additional high-pass filter of 0.15 Hz and/or a low-pass filter of 8.5 to 45 Hz. The P300 amplitude and latency were measured at the peak between 250 and 600 ms at the Pz electrode. Details of EEG collection at each research centre are listed in Table S2.

Table S2. Parameters for EEG acquisition and processing.

| Research centre | Number of scalp sites | Reference | Sampling rate (Hz) | EEG pass filtering (Hz) | Artifact correction | P300 window (ms) | Processing pass filtering (Hz) |
| --- | --- | --- | --- | --- | --- | --- | --- |
| Heidelberg | 20 | Linked mastoids | 400 | Low pass filter = 70 | Regression-based weighting coefficients | 270 - 470 | - |
| London | 17 | Left earlobe | 500 | 0.03 - 120 | Regression-based weighting coefficients | 280 - 500 | 0.03 - 45 |
| Perth | 20 | Left and right earlobes and mastoids | 200 | 0.05 - 30 | Regression-based weighting coefficients and manual check | 250 - 550 | Low pass filter = 40 |

MRI data collection and processing

The procedure of MRI collection and processing varied across research centres. In summary, images were acquired by 1.5 to 3 T scanners, with flip angel = 9 to 45°, repetition time (TR) = 7.92 to 2,250 ms, and echo time (TE) = 2.3 to 5.8 ms. Voxels were analysed using automatic labelling tools or region of interest analysis based on previous literature. The lateral ventricular volume included the body of the lateral ventricle and its frontal, occipital, and temporal horns. See Table S3 or previous publications for details at each research centre.^43–58^

Table S3. Parameters for MRI acquisition and processing.

| Research center | Scanner | Acquisition sequence | Repetition time (TR; ms) | Echo time (TE; ms) | Flip angle (°) | Processing software |
| --- | --- | --- | --- | --- | --- | --- |
| Edinburgh | 1 T Siemens Magnetom | Magnetisation prepared rapid acquisition gradient echo (MPRAGE). | 10 | 4 | 12 | Analyze |
| Heidelberg | 1.5 T (Tesla) Phillips | Magnetisation prepared rapid acquisition gradient echo (MPRAGE) | 11.4 | 4.4 | 15 | Analyze |
| London | 1.5 T General Electric (USA) Signa System | Spoiled gradient recall (SPGR) echo | 14.7 | 3.7 | 20 | MEASURE |
|  |  |  | 9.8 | 2.3 | 20 |  |
|  |  |  | 13.1 | 5.8 | 20 |  |
| Santander | 1.5 T General Electric Signa System | Spoiled gradient-recalled acquisition in the steady state (GRASS) (SPGR) | 24 | 5 | 45 | BRAINS2 |
| The Netherlands | 3 T Siemens | Modified driven equilibrium Fourier transform (MDEFT) | 7.92 | 2.4 | 15 | Freesurfer |
|  |  | Magnetization prepared rapid acquisition gradient echo (MPRAGE) | 2250 | 2.6 | 9 |  |
|  | 1.5 T Philips NT | Fast field echo (FFE) | 30 | 4.6 | 30 | A histogram method validated previously |

Genotyping, quality control, and imputation

6,935 blood samples were sent to the Wellcome Trust Sanger Institute (Cambridge, UK). They were processed in a 96-well plate format with each carrying a positive and negative control. A PicoGreen assay (Invitrogen, Life Technologies, Grand Island, New York) and an aliquot assayed by agarose gel electrophoresis were used to quantify DNA concentrations. To pass quality control, the DNA concentration should not be degraded and be at least 50 ng/mL.

30 SNPs including sex chromosome markers were firstly typed on the Sequenom platform and then entered whole genome genotyping. 347 samples were excluded because of degraded or insufficient DNA or incorrect sex classification. Subsequently the samples left were sent to the Affymetrix Services Laboratory (www.affymetrix.com).

Samples were genotyped using the Genome-wide Human SNP Array. Genotypes were called using the CHIAMO algorithm modified for use with the Affymetrix 6.0 genotyping array ^59,60^. Approximately 30% of the samples showed poor signal-to-noise ratio in the genotyping array and thus were excluded. The excluded samples were randomly distributed across groups (32% of patients, 30% of relatives and 30% of controls; χ2 (2 df) = 3.2; *p* = 0.20).

Quality control of the genotyped data was conducted using PLINK.^64^ 11,610 SNPs were excluded due to a study-wide missing rate > 5%. 26,858 SNPs were excluded due to four or more Mendelian inheritance errors identified by PEDSTATS^61^. 2,404 SNPs were excluded due to departure from Hardy-Weinberg equilibrium (*p* < 10^–6^), and 145,097 SNPs were excluded due to a MAF < 0.02. 38,895 SNPs from the sex chromosomes or mitochondrial DNA were also excluded. 9,499 were excluded via visual inspection of genotyping intensity plots generated by Evoker.^62^

214 samples were excluded as they had more than 2% missing data of all SNPs. 70 samples were excluded because of divergent genome-wide heterozygosity identified by PLINK (inbreeding coefficients were F > 0.076 or F < -0.076).^64^ A thinned subset of 71,677 SNPs were used to infer chromosomal sharing using LDAK.^63^ 70 pairs of duplicates and monozygotic twins were identified by an identity by descent > 95%, and one in each pair with more complete data was kept.

Quality controlled genotypes were uploaded to the Sanger Imputation Server (https://imputation.sanger.ac.uk).^65^ Pre-phasing and imputation were conducted according to the EAGLE2/PWBT pipeline based on the Haplotype Reference Consortium panel (r1.1).^66,67^ The imputed genotypes were converted to best-guess format using a hard-call threshold of 0.8 and SNPs with an INFO score < 0.8 were excluded. We also conducted additional quality control on the imputed genotypes using PLINK,^64^ by excluding SNPs with a missing rate > 5%, a MAF < 1%, departure from the Hardy-Weinberg equilibrium (p < 1e^-6^), Mendelian error rate > 10%, or cases versus controls data missingness significance < 5e^-6^. Samples were excluded due to a missing rate > 5%, a Mendelian error rate > 5%, or an inbreeding coefficient > 0.1. Duplicates or twins were identified by LDAK and one of each pair was removed.^63^ A total of 6,215,801 SNPs and 4,835 samples remained after quality control.

Population structure and relatedness inference

To account for familial relatedness and population structure in the sample, we used the GENESIS R/Bioconductor package to generate a kinship matrix and conduct a principal component (PC) analysis.^71,72^ Based on the genotyped data that passed quality control, an unadjusted kinship matrix was firstly generated using KING-robust 2.2.5.^73^ The kinship coefficient is defined as the probability that two alleles selected at random from two individuals are identical by descent.^73^ The kinship coefficient for monozygotic twins is expected to be 2ˆ(−2/2) = 0.5, and that for first-degree relatives is 2ˆ(−4/2) = 0.25.^73^ Thus, 2ˆ(−3/2) = 0.354 was used as the threshold differentiating monozygotic twins and first-degree relatives, and the same rule applies to second-degree, third-degree, and more distant relationships.^73^

Based on the output from KING-robust, we then conducted a principal component analysis using the PC-AiR package.^71^ Genotyped data were filtered and pruned using the SNPRelate package in R with a MAF threshold = 0.05, a linkage disequilibrium threshold = √0.1, and a maximum sliding window of 10^6^ bp ^74^. Any pair of individuals with a kinship coefficient greater than 2ˆ(11/2) = 0.022 (less than fourth-degree relatives) was defined as related, and any pair of individuals with a kinship coefficient less than −0.022 was defined as unrelated.

Finally, we performed a PC-Relate analysis to estimate the familial relatedness adjusted for ancestral background.^75^ The unrelated subset of the sample and the first four ancestrally representative PCs estimated in the previous steps were used to estimate the relationships. In this way, PC-Relate generated a kinship matrix with an adjusted kinship coefficient between each pair of individuals. We set any adjusted kinship coefficients < 0.022 to 0 to improve the processing speed in the following regressions.

***Associations between endophenotypes/case-control status and polygenic risk scores***


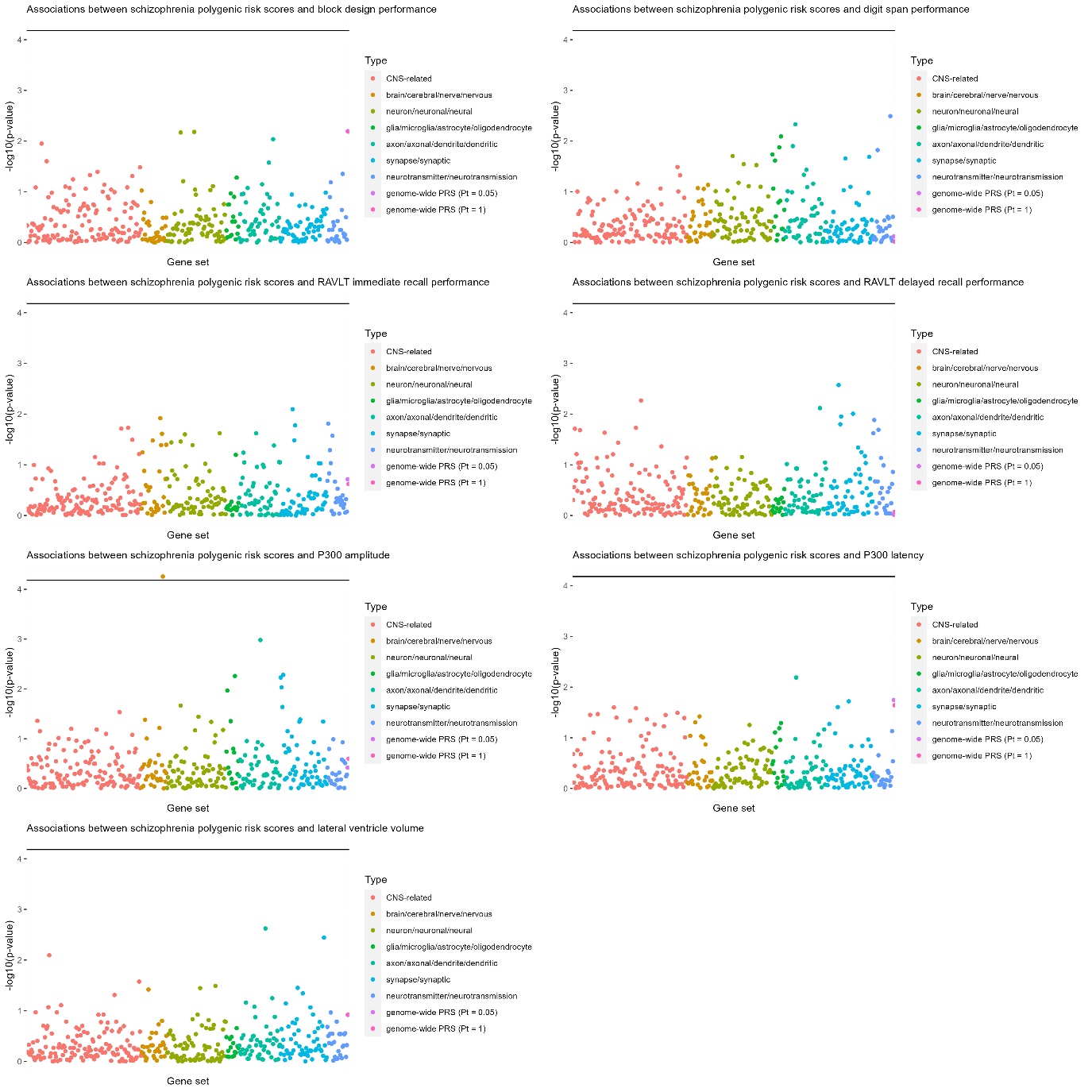


Figure S1. Associations between endophenotypes and schizophrenia polygenic risk scores. Gene-set specific polygenic risk scores are grouped by the search terms they contain. CNS-related polygenic risk scores were generated based on custom annotated gene sets from previous publications.^76–78^ On the x-axis, gene sets from the same source were arranged in descending order of the number of SNPs included in each polygenic risk score. CNS, central nervous system. PRS, polygenic risk score. Pt, p-value threshold. RAVLT, Rey auditory verbal learning test.


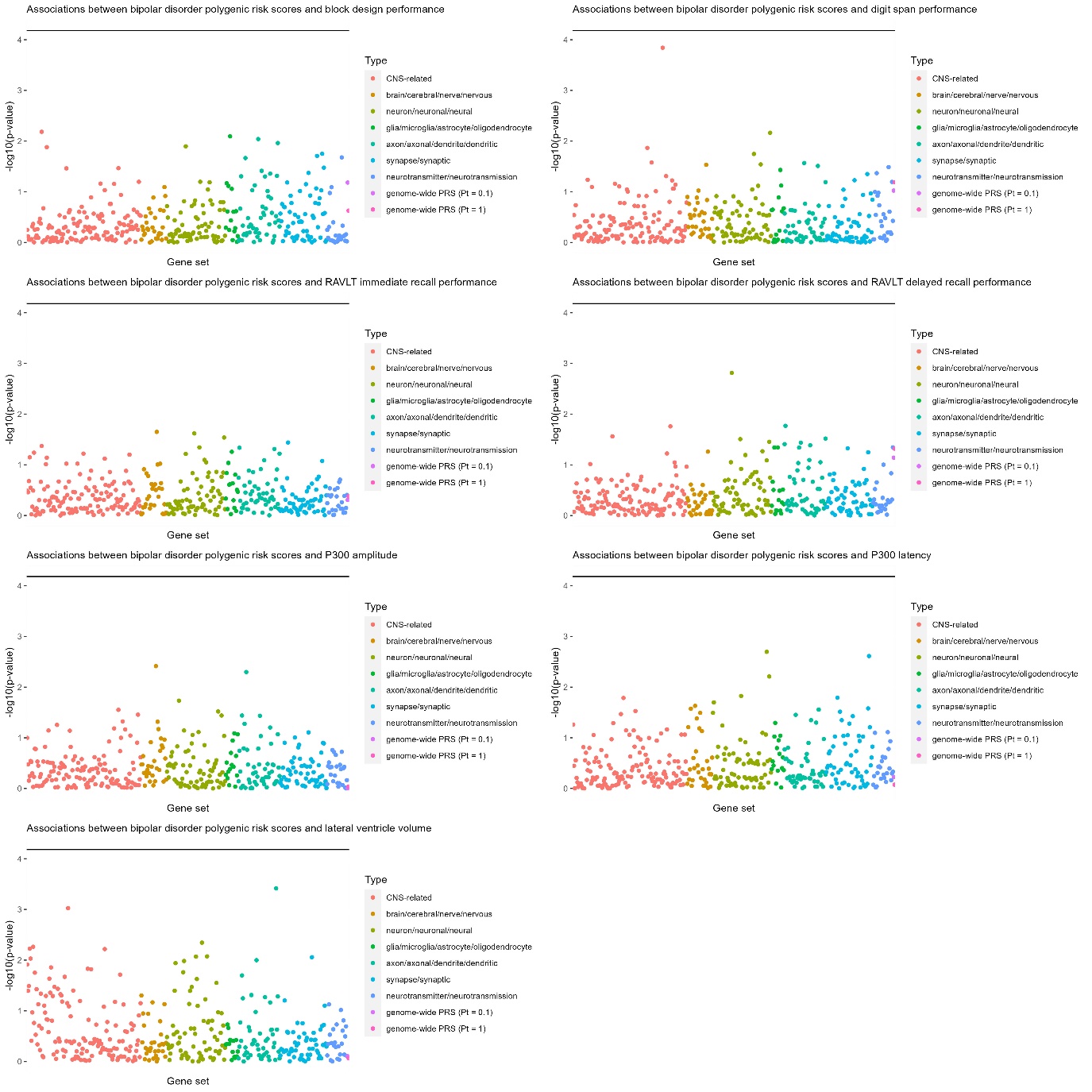


Figure S2. Associations between endophenotypes and bipolar disorder polygenic risk scores. Gene-set specific polygenic risk scores are grouped by the search terms they contain. CNS-related polygenic risk scores were generated based on custom annotated gene sets from previous publications.^76–78^ On the x-axis, gene sets from the same source were arranged in descending order of the number of SNPs included in each polygenic risk score. CNS, central nervous system. PRS, polygenic risk score. Pt, p-value threshold. RAVLT, Rey auditory verbal learning test.


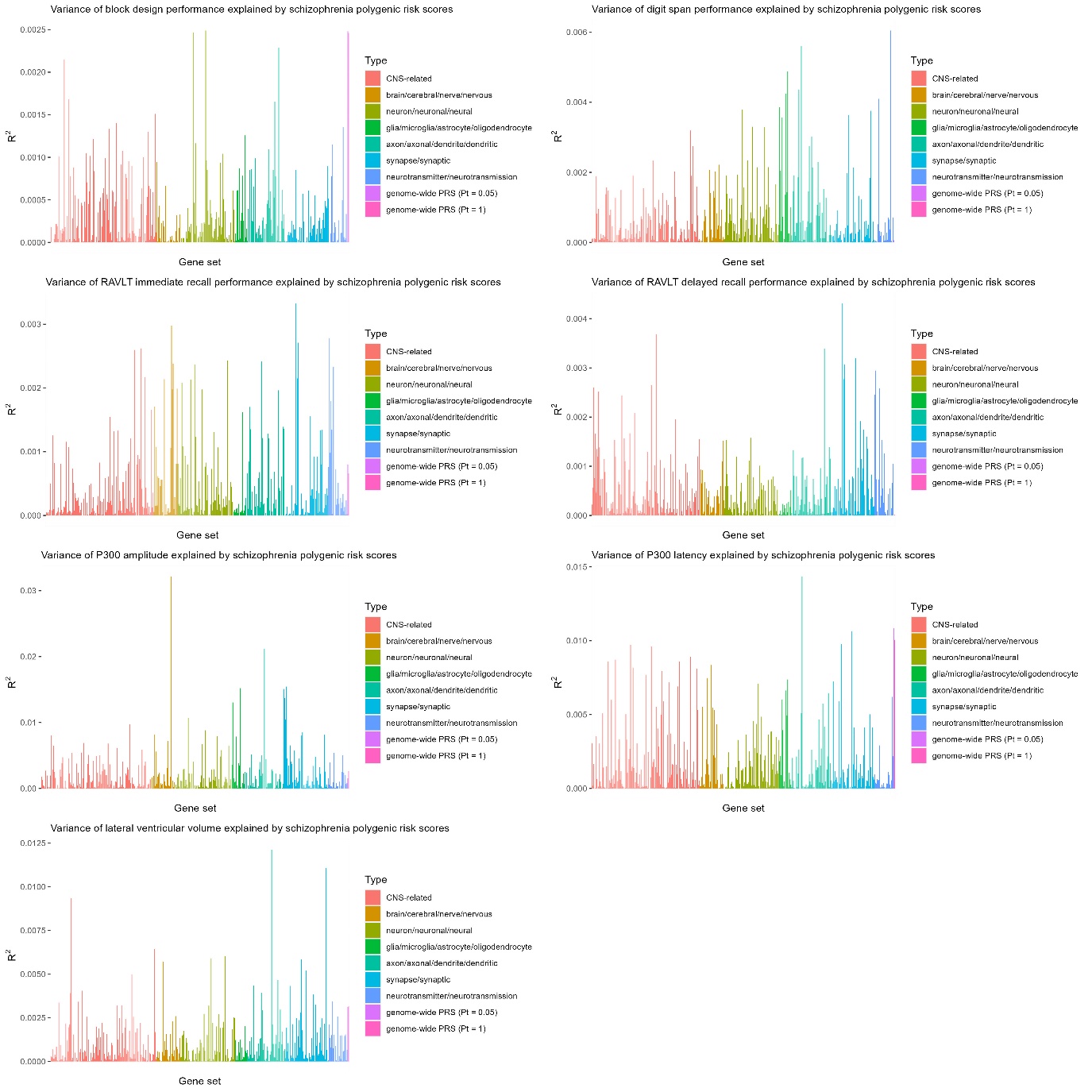


Figure S3. Variance of endophenotypes explained by schizophrenia polygenic risk scores. Gene-set specific polygenic risk scores are grouped by the search terms they contain. CNS-related polygenic risk scores were generated based on custom annotated gene sets from previous publications.^76–78^ On the x-axis, gene sets from the same source were arranged in descending order of the number of SNPs included in each polygenic risk score. PCNS, central nervous system. PRS, polygenic risk score. Pt, p-value threshold. RAVLT, Rey auditory verbal learning test.


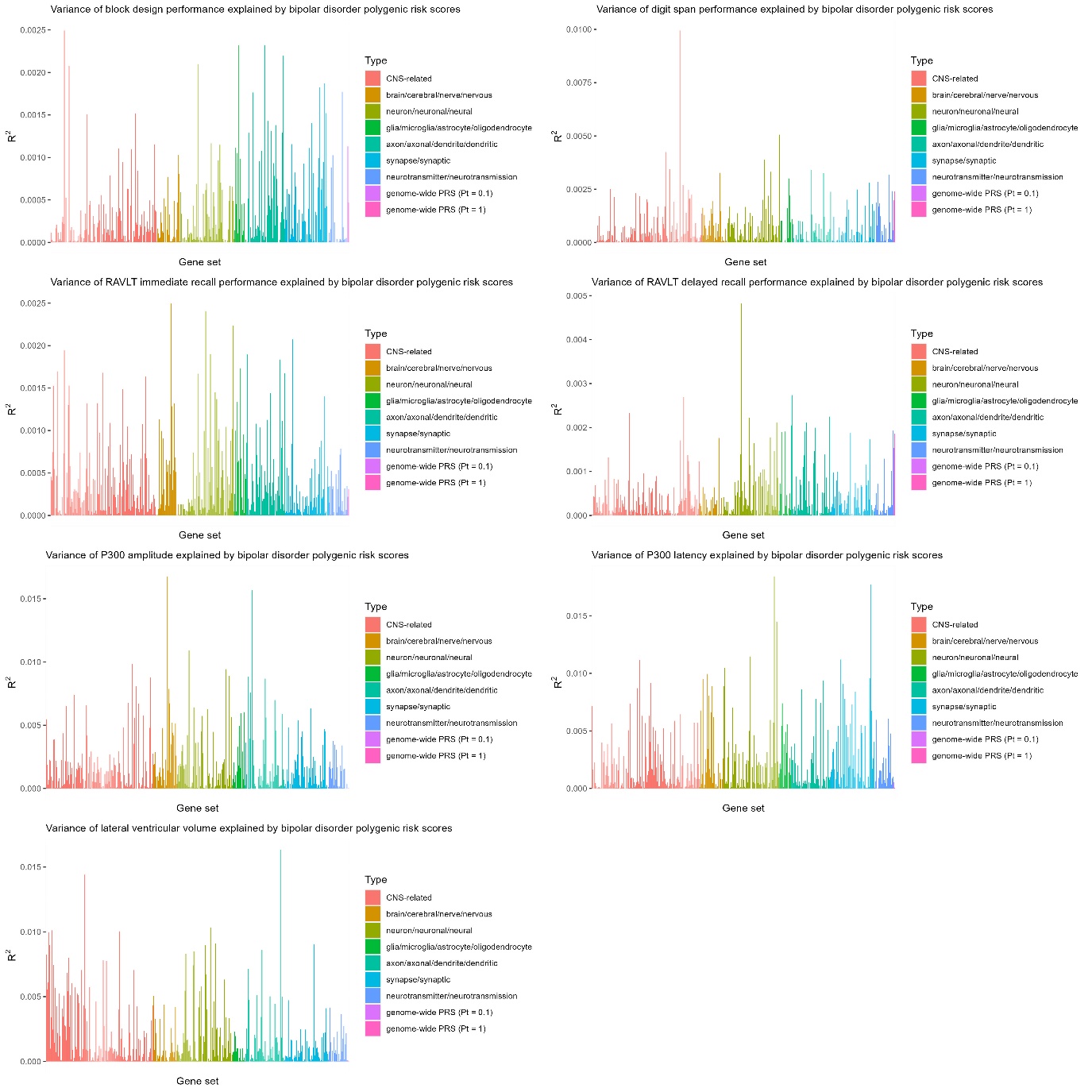


Figure S4. Variance of endophenotypes explained by bipolar disorder polygenic risk scores. Gene-set specific polygenic risk scores are grouped by the search terms they contain. CNS-related polygenic risk scores were generated based on custom annotated gene sets from previous publications.^76–78^ On the x-axis, gene sets from the same source were arranged in descending order of the number of SNPs included in each polygenic risk score. CNS, central nervous system. PRS, polygenic risk score. Pt, p-value threshold. RAVLT, Rey auditory verbal learning test.

***Association between P300 amplitude and schizophrenia polygenic risk score of forebrain regionalization***

As validation, we checked if the association between the P300 amplitude and the schizophrenia polygenic risk score of forebrain regionalization was consistent across three clinical groups. The direction of the association was consistent in all groups (Figure S5). We found that reduced P300 amplitudes were associated with higher schizophrenia polygenic risk scores of forebrain regionalization in both patients (mean difference: -1.15 µV; 95% CI: -1.89 to -0.40 µV; *p* = 0.003) and controls (mean difference: -1.23 µV; 95% CI: -2.38 to -0.09 µV; *p* = 0.037), although this association did not reach the nominal significance level in relatives (mean difference: -0.90 µV; 95% CI: -2.00 to 0.20 µV; *p* = 0.113).

Figure S5. Association between P300 amplitude and the schizophrenia polygenic risk score of forebrain regionalization across three groups.

Genes included in the forebrain regionalization gene sets are *ADGRG1, AXIN1, BMP2, BMP4, DMRTA2, EMX1, EMX2, EOMES, FEZF1, FEZF2, FGF8, GLI3, GSX2, LHX1, LHX2, NKX2-1, PAX6, PGAP1, SHH, SIX3, TRA2B, TTC21B, WNT1, WNT2B, and WNT7B.* Among those genes, *EMX1* contained a locus that reached genome-wide significance in the latest GWAS on schizophrenia.^4^ Therefore, we conducted an additional analysis testing the association between the P300 amplitude and the partitioned schizophrenia polygenic risk score restricted to the region of *EMX1*, which also yielded a significant association at the nominal significance level (mean difference per SD increase in polygenic risk score: −0.66 µV, 95% CI: −1.27 to −0.05, *p* = 0.033).

***Influence of number of SNPs included in polygenic risk scores on associations***

We examined the Spearman rank-based correlations between the number of SNPs in gene-set specific polygenic risk scores and the level of significance (-log10(p-value)) for their associations with endophenotypes. We found significant correlations between the number of SNPs included in the schizophrenia polygenic risk scores and the -log10(p-value) for RAVLT immediate recall performance (ρ = -0.11, *p* = 0.027) and P300 latency (ρ = -0.11, *p* = 0.032). There were also significant correlations between the number of SNPs included in the bipolar disorder polygenic risk scores and the -log10(p-value) for block design task performance (ρ = -0.14, *p* = 0.006) and lateral ventricular volume (ρ = 0.16, *p* = 0.002). All other correlations were not significant, and the coefficients were very low (|ρ| < 0.1). Considering the directions of the correlations were different and the coefficients were small, we believe there were no consistent patterns for the correlations between the number of SNPs in gene-set specific polygenic risk scores and the level of significance for their associations with endophenotypes. By contrast, for case-control status, the -log10(p-value) was consistently and positively correlated with the number of SNPs included in both schizophrenia (ρ = 0.74, *p* < 0.001) and bipolar disorder gene-set specific polygenic risk scores (ρ = 0.45, *p* < 0.001). This shows that the more SNPs a polygenic risk score included, the more significant its association with case-control status was.
